# Isolation and Characterization of Isofraxidin 7-*O*-(6′-*O*-*p*-Coumaroyl)-*β*-glucopyranoside from* Artemisia capillaris* Thunberg: A Novel, Nontoxic Hyperpigmentation Agent That Is Effective In Vivo

**DOI:** 10.1155/2017/1401279

**Published:** 2017-05-24

**Authors:** Soon-Ho Yim, Nadia Tabassum, Woong-Hee Kim, Haaglim Cho, Ji-Hyung Lee, Galzad J. Batkhuu, Hyun Jung Kim, Won Keun Oh, Da-Woon Jung, Darren R. Williams

**Affiliations:** ^1^Department of Pharmaceutical Engineering, Dongshin University, Jeonnam, Republic of Korea; ^2^New Drug Targets Laboratory, School of Life Sciences, Gwangju Institute of Science and Technology, Gwangju 500-712, Republic of Korea; ^3^School of Engineering and Applied Sciences, National University of Mongolia, Ulaanbaatar, Mongolia; ^4^College of Pharmacy and Natural Medicine Research Institute, Mokpo National University, Jeonnam, Republic of Korea; ^5^College of Pharmacy, Seoul National University, Seoul, Republic of Korea

## Abstract

Abnormalities in skin pigmentation can produce disorders such as albinism or melasma. There is a research need to discover novel compounds that safely and effectively regulate pigmentation. To identify novel modulators of pigmentation, we attempted to purify compounds from a bioactive fraction of the Korean medicinal plant* Artemisia capillaris* Thunberg. The novel compound isofraxidin 7-*O*-(6′-*O*-*p*-coumaroyl)-*β*-glucopyranoside (compound** 1**) was isolated and its pigmentation activity was characterized in mammalian melanocytes. Compound** 1** stimulated melanin accumulation and increased tyrosinase activity, which regulates melanin synthesis. Moreover, compound** 1** increased the expression of tyrosinase and the key melanogenesis regulator microphthalmia-associated transcription factor (MITF) in melanocytes. Compared to the parent compound, isofraxidin, compound** 1** produced greater effects on these pigmentation parameters. To validate compound** 1** as a novel hyperpigmentation agent in vivo, we utilized the zebrafish vertebrate model. Zebrafish treated with compound** 1** showed higher melanogenesis and increased tyrosinase activity. Compound** 1** treated embryos had no developmental defects and displayed normal cardiac function, indicating that this compound enhanced pigmentation without producing toxicity. In summary, our results describe the characterization of novel natural product compound** 1 **and its bioactivity as a pigmentation enhancer, demonstrating its potential as a therapeutic to treat hypopigmentation disorders.

## 1. Introduction

Human skin is exposed to numerous environmental stresses, such as mutagenic ultraviolet radiation. Mutations or dysregulation of the genes that regulate pigmentation can produce hypopigmentation disorders, such as albinism or vitiligo [1, 2]. Hypopigmented lesions may disfigure the skin, produce psychosocial problems, and increase the probability of developing skin cancer [3].

Small molecule compounds that increase melanin synthesis have the potential to treat hypopigmentation disorders [4]. Numerous pigmentation enhancing agents have been discovered, such as diacylglycerols, 3-isobutyl-1-methylxanthine (IBMX), and dimethylsulfoxide (DMSO). However, their side effects can be a major concern, including the propensity to induce tumors [5, 6]. Therefore, there is a need to discover new compounds that increase skin pigmentation.

**Figure 1:**
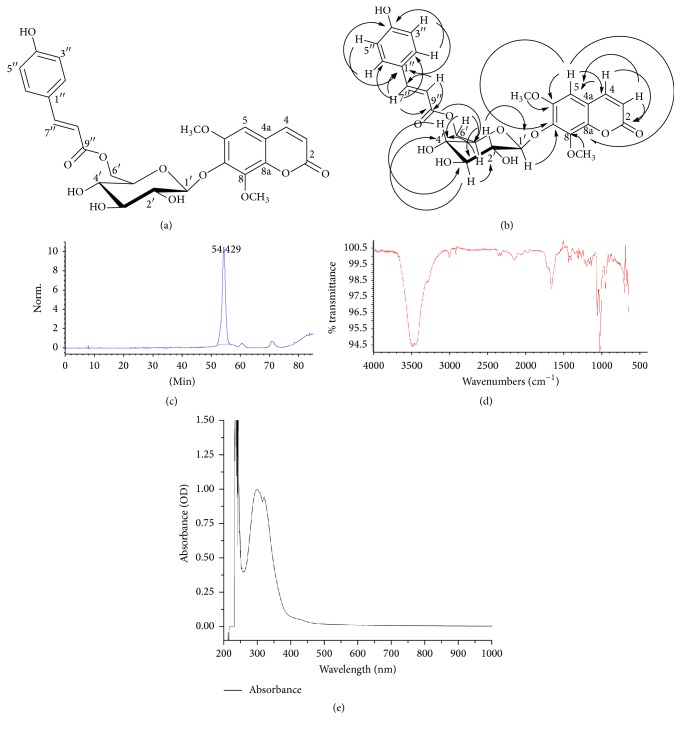
Characteristics of compound** 1** purified from* A. capillaris*. (a) Chemical structure of compound** 1**. (b) Key HMBC correlations for compound** 1**. (c) HPLC chromatogram at 280 nm of compound** 1**. (d) Infrared (IR) spectrum and (e) absorption UV spectrum of compound** 1**.

In this study, we identified a new pigmentation-regulating compound in the plant* Artemisia capillaris* Thunberg (*A. capillaris*; Oriental wormwood). The* Artemisia* genus has been used as a herbal medicine for a range of disorders, including inflammation, microbial infections, cancer, and malaria [7]. Extracts derived from* Artemisia capillaris* Thunberg have been shown to produce a number of biological effects, such as antiplatelet aggregation activity, hepatocyte protection, anti-inflammatory activity, antioxidant activity, antitumor activity, and antiobesity action [8–12]. Using an active fraction from* A. capillaris*, we purified a novel enhancer of pigmentation: isofraxidin 7-*O*-(6′-*O*-*p*-coumaroyl)-*β*-glucopyranoside (compound** 1**; Figure 1(a)). Compound** 1** was found to produce hyperpigmentation effects in vivo, suggesting that it can be developed as a pharmaceutical/cosmetic agent to treat skin disorders resulting from reduced pigmentation.

## 2. Materials and Methods

### 2.1. General Experimental Procedures

Optical rotation was measured with a JASCO DIP-1000 digital polarimeter. UV spectra were recorded with a JASCO V-530 UV/Vis spectrophotometer (Jasco Corp., Japan) and IR spectra were obtained using a JASCO FT/IR-300E spectrometer (Jasco Corp., Japan). ^1^H and ^13^C NMR spectra were recorded using a Varian VNMRS 600 MHz NMR spectrometer (Varian, Inc., USA) for 1D and 2D NMR experiments in CD_3_OD [tetramethylsilane (TMS) was used as the internal standard]. The chemical shifts (*δ*) were expressed as parts per million (ppm) and the coupling constants (*J*) were expressed in Hz. Mass spectra were measured on a JMS-700 (Jeol, Japan) and Varian 1200, Platform II (Varian, USA) spectrometers. High resolution mass (ESI-MS) and LCMS/MS spectra were measured with a Waters Synapt High Definition Mass Spectrometry/time-of-flight mass spectrometer, using the negative electrospray ion mode.

### 2.2. Plant Materials

The active fraction (ACMF09) from* Artemisia capillaris* Thunberg was provided by Professor Soon-Ho Yim, Dongshin University, Jeonnam, Republic of Korea.

### 2.3. Reagents

IBMX, forskolin, phenylthiourea (PTU), sodium hydroxide (NaOH), DMSO,* L-3,4*-dihydroxyphenylalanine (L-DOPA), caffeine powder, staurosporine, 3-(4,5-dimethylthiazol-2-yl)-2,5-diphenyltetrazolium bromide (MTT), and CellLytic™ lysis buffer were purchased from Sigma (St Louis, MO, USA). Isofraxidin was purchased from ChemFaces Biochemical (Hubei, China).

### 2.4. Cell Culture

Murine melanoma B16F10 melanocytes were supplied by the American Type Culture Collection (ATCC, Manassas, VA) and cultured in DMEM containing 10% FBS and 1% penicillin-streptomycin mixture (Gibco, USA). Cells were cultured in a 37°C humidified incubator with 5% CO_2_.

### 2.5. Determination of Melanin Content in B16F10 Melanocytes

The melanocytes were seeded in a 6-well plate at a density of 2 × 10^5^ cells/well. 24 h later, the cells were treated with compound of interest for 48 h. Cells were washed with PBS and lysed with 4°C CellLytic buffer, followed by centrifugation at 13000 rpm for 10 min at 4°C. The pellet was used for melanin content analysis by two washes with ethanol : ether (1 : 1 volume) and dissolving in 200 *μ*L 1 N NaOH containing 10% DMSO, with heating at 80°C for 1 h. 100 *μ*L aliquots of the solution were measured for melanin content by reading the absorbance at 400 nm using a microplate reader (VersaMax™; Molecular Devices Corporation, California, USA), as previously described.

### 2.6. Measurement of Melanin Secreted into the Culture Media

Based on the previously published protocol [15]. B16F10 melanocytes were seeded in a 24-well plate at a density of 5 × 10^4^ cells/well using 500 *μ*L culture media. 24 h later, the culture media were replenished and cells were treated with the compound of interest for 60 h. The culture media were collected and melanin levels were measured at an absorbance wavelength of 475 nm using a microplate reader (VersaMax; Molecular Devices Corporation, California, USA).

### 2.7. Determination of Tyrosinase Activity in Melanocytes

Tyrosinase activity in the compound treated melanocytes was measured as previously described [16]. In brief, B16F10 melanocytes were seeded in a 6-well plate at 2 × 10^5^ cells/well. 24 h later, the cells were treated with compound of interest for 48 h. The cells were rinsed with PBS and lysed with CellLytic buffer at 4°C. Cell extracts were then centrifuged at 13000 rpm for 10 min at 4°C. The protein concentration was adjusted using the Bradford assay and 100 *μ*L lysate containing 40 *μ*g protein was transferred into a 96-well plate. 100 *μ*L of 5 mM L-DOPA was added, followed by incubation at 37°C for 60 min. Dopachrome formation was measured at 475 nm with a plate reader.

### 2.8. Mushroom Tyrosinase Assay

The effect of the compounds on mushroom tyrosinase activity was measured using the assay of Zhang et al. [17] with the following modifications. Mushroom tyrosinase was dissolved in 50 mM potassium phosphate buffer (pH 6.5) to a concentration of 500 units/mL. 550 *μ*L of 50 mM potassium phosphate and 50 *μ*L of the mushroom tyrosinase solution were combined with the test samples in a microfuge tube and incubated for 5 min at room temperature. 100 *μ*L of 1.5 mM* L*-tyrosine was added and the solution was transferred to a 96-well plate. Formation of the dopachrome product was determined by measuring the absorbance at 490 nm using a microplate reader.

### 2.9. Purification of Compound** 1**

The active fraction of the MeOH extract of* A. capillaris* was separated using a RP-18 column with a gradient of H_2_O-MeOH (60 : 40 v/v) for 50 min and then changed to 0 : 100 for 20 min to yield compound** 1** (2 mg). 

*Isofraxidin 7-O-(6*′*-O-p-Coumaroyl)-β-glucopyranoside*. Brownish amorphous powder; UV *λ*_max_ (H_2_O) (log⁡*ε*) 300, 304, 322 nm; IR (KBr) *ν*_max_: 3500, 1675, 1011 cm^−1^; ^1^H NMR (in CD_3_OD, 600 MHz)*δ* 7.64 (1H, d,* J* = 9.6 Hz, H-4), 7.37 (1H, d,* J* = 15.9 Hz, H-7′′), 7.32 (2H, d,* J* = 8.7 Hz, H-2′′/H-6′′), 6.90 (1H, s, H-5), 6.80 (2H, d,* J* = 8.7 Hz, H-3′′/H-5′′), 6.16 (1H, d,* J* = 9.6 Hz, H-3), 6.10 (1H, d,* J* = 15.9 Hz, H-8′′), 5.20 (1H, d,* J* = 7.5 Hz, H-1′), 4.39 (1H, dd,* J* = 11.4, 7.2 Hz, H-6′), 4.34 (1H, dd,* J* = 11.4, 2.0 Hz, H-6′), 3.99 (3H, s, 8-OMe), 3.88 (3H, s, 6-OMe), 3.55 (1H, dd,* J* = 9.0, 7.5 Hz, H-2′), 3.50 (1H, m, H-5′), 3.47 (1H, t,* J* = 9.0 Hz, H-3′), 3.40 (1H, t,* J* = 9.0 Hz, H-4′); ^13^C NMR (in CD_3_OD, 125 MHz) *δ* 168.7 (C-9′′), 162.7 (C-2), 161.5 (C-4′′), 151.8 (C-6), 146.5 (C-7′′), 145.7 (C-4), 144.0 (C-8a), 143.1 (C-7), 142.7 (C-8), 131.3 (C-2′′/C-6′′), 127.1 (C-1′′), 117.1 (C-3′′/C-5′′), 116.9 (C-4a), 115.7 (C-3), 114.9 (C-8′′), 105.9 (C-5), 103.9 (C-1′), 77.9 (C-3′), 75.9 (C-5′), 75.7 (C-2′), 72.2 (C-4′), 64.4 (C-6′), 62.5 (8-OMe), 57.2 (6-OMe); HRESI-MS* m/z* 529.1346 [M−H]^−^ (C_26_H_25_O_12_).

### 2.10. Quantitative Real-Time PCR Analysis

B16F10 melanocytes were treated with test compounds for 48 h. Total RNA was extracted using the TRI-Solution™ according to the manufacturer's instructions and quantified using a NanoDrop 2000 spectrophotometer (NanoDrop Technologies). cDNA synthesis was carried out from 1 *μ*g RNA using AccuPower® PCR PreMix (Bioneer) following the manufacturer's recommendation. mRNA expression of the MITF gene, tyrosinase gene, and TRP-1 was quantified using a Power SYBT® Green PCR Master Mix (Applied Biosystems). mRNA levels were normalized with *β*-actin and fold change of expression was calculated with the* ΔΔ*CT method. The primer sequences were as follows: mouse tyrosinase forward 5′-TACTTGGAACAAGCCAGTCGTATC-3′, reverse 5′-ATAGCCTACTGCTAAGCCCAGAGA-3′; mouse TRP-1 forward 5′-AAACCCATTTGTCTCCCAATGA-3′, reverse 5′-CGTTTTCCAACGGGAAGGTA-3′; mouse MITF forward 5′-GGACTTTCCCTTATCCCATCCA-3′, reverse 5′-GCCGAGGTTGTTGGTAAAGGT-3′. The PCR conditions were 95°C for 2 min followed by 40 cycles of 95°C for 30 s, 60°C for 1 min, and 72°C for 1 min followed by a final 30 sec extension at 72°C. Data were analyzed using the Stepone™ software v2.3 (Applied Biosystems).

### 2.11. MTT Assay for Cell Viability

Cell viability was assessed using the MTT assay, as previously described [18]. B16-F10 melanocytes were seeded into a 96-well plate at the density of 5 × 10^3^ cells/well for 12 h. Cells were treated with compound or extract for 48 h.

### 2.12. Maintenance of Zebrafish Fish

The experimental protocols involving zebrafish were approved by the Animal Care and Use Committee of the Gwangju Institute of Technology, Republic of Korea. All methods that utilized zebrafish were carried out in accordance with relevant guidelines and regulations provided by the Gwangju Institute of Science and Technology. Adult zebrafish* (Danio rerio)* were purchased from a commercial outlet (Lotte Mart, Republic of Korea). 10–15 fishes were maintained in a 5 L acrylic tank under the following conditions: water temperature 28.5°C with a 14/10 h light/dark cycle. Zebrafish were fed twice daily with live brine shrimps* (Artemia salina)*. Fish breeding was carried out using the standard protocol [19] at 9.30 AM in the morning. Embryos were collected within 30 min of spawning.

### 2.13. Compound Treatment and Evaluation of Pigmentation Phenotype in Zebrafish Embryos

Zebrafish embryos were maintained in 100 mm^2^ Petri dishes using embryo media (5 mM NaCl, 0.17 mM KCl, 0.33 mM CaCl_2_·2H_2_O, and 0.33 mM MgSO_4_·7H_2_O, to provide a 60x stock solution) at a density of 70–80 embryos/dish. For compound treatment, embryos were placed in a 96-well plate (3 embryos/well in 200 *μ*L embryo media). Compounds were dissolved in 0.1% DMSO and embryos were treated from 9 to 72 hpf (63 h exposure). The media and embryos were gently stirred and media/compound was replenished daily to ensure an even compound distribution. Effects on the pigmentation were imaged by stereomicroscopy (LEICA DFC425 C; 100x magnification). In all experiments, 75 *μ*M PTU was used as a positive control to ablate zebrafish pigmentation without interfering with developmental processes [20]. For microscopy, the embryos were dechorionated using forceps, anesthetized in tricainemethanesulfonate solution (Sigma), and mounted using 3% methyl cellulose. Melanocyte size and pigmentation intensity were calculated using the Image J software (National Institutes of Health, USA).

### 2.14. Measurement of Melanin Content and Tyrosinase Activity in Zebrafish

Tyrosinase activity was measured as described previously [21] with the following minor modifications: 40 zebrafish embryos were treated with compound of interest from 9 to 48 hpf, followed by homogenization by sonification in CellLytic buffer (pulse on: 10 s, pulse off: 5 s, 8 min in 150 *μ*L buffer). The lysate was centrifuged at 13000 rpm for 10 min. the supernatant was collected and protein was quantified using the Bradford assay. 250 *μ*g total protein in 100 *μ*L buffer was transferred to a 96-well plate. 100 *μ*L 5 mM L-DOPA was added, followed by incubation for 60 min at 37°C. The absorbance was determined at 475 nm using a microplate reader. The blank sample reading was deleted from each absorbance value, with the final activity expressed as a percentage of the water control.

Melanin content in the zebrafish was measured using the previously described protocol [21]. In brief, protein extracts were prepared at 48 hpf using the same methodology as the tyrosinase assay. After centrifugation, the pellet was collected and dissolved in 200 *μ*L 1 N NaOH with 10% DMSO for 80°C for 60 min, followed by vortexing. Absorbance was measured at 400 nm using a microplate reader.

### 2.15. Measurement of Zebrafish Heart Rate

To determine the toxicity of test compounds, the heart rate of the atrium and ventricle was measured for 3 min at 48 hpf to assess toxicity, as previously described [22] using a Zeiss Stemi 2000-C stereomicroscope.

### 2.16. Melanocyte Counting Assay

To count melanocytes, embryos were exposed to the fish room lighting. This induces contraction of the melanin within the melanocytes [23]. Embryos were then fixed in 4% paraformaldehyde and imaged using stereomicroscopy. Melanocytes were counted in the head region of the embryos.

### 2.17. Statistical Analysis

Data were evaluated for significance using Student's* t*-test (Microsoft Excel 2016). A *p* value of less than 0.05 was considered to be significant. Data are expressed as the means ± SEM of three independent experiments, unless otherwise indicated.

## 3. Results

### 3.1. Purification and Isolation of Compound** 1** from the* A. capillaris* Plant Extract

An active fraction from* A. capillaris* was previously shown to regulate melanin synthesis [24]. Preparative reversed-phase HPLC was performed to purify additional compounds from this active fraction. A 2.0 mg compound (termed compound** 1**; Figures 1(a) and 1(b)) was purified. The chromatogram obtained for compound** 1** extracted with a retention time 54.428 min is shown in Figure 1(c). Compound** 1** was identified and characterized on the basis of complementary information provided by ESI-MS detectors. The structure of the isolate was identified by spectroscopic analysis (^1^H NMR), as a novel compound. The IR and UV spectra for purified compound** 1** are shown in Figures 1(d) and 1(e). This compound was identified and characterized using complementary information provided by the ESI-MS detectors. The appearance of isolated compound** 1** was a pale brown, amorphous powder. Compound** 1** was assigned the molecular formula, C_26_H_25_O_12_, based on the HRESIMS spectra of the compound obtained in the negative ion mode (*m/z* 529.1346 [M−H]^−^.calcd for C_26_H_25_O_12_). Compound** 1** exhibited UV absorption bands at 300, 304, and 322 nm with IR absorption bands corresponding to hydroxy (3500 cm^−1^) and carbonyl (1675 cm^−1^). The 1D NMR spectra of compound** 1** exhibited signals for a coumarin moiety, a* p*-coumaroyl moiety, and a *β*-glucopyranose unit. Compound** 1** showed two doublets at *δ*_H_ 7.64 (1H, d,* J* = 9.6 Hz, H-4), *δ*_H_ 6.16 (1H, d,* J* = 9.6 Hz, H-3), and a carbonyl ester at *δ*_C_ 162.7 (C-2) ascribable to the coumarin moiety and exhibited the presence of one *β*-glucopyranose unit from the anomeric signals at *δ*_H_ 5.20 (1H, d,* J* = 7.8 Hz, H-1′) including hydroxyl methine signals at *δ*_H_ 3.40~3.55 with vicinal coupling constants (*J* = 9.0 Hz) between two axial hydrogens. The signals of* p*-coumaroyl moiety were assigned to symmetric doublets at *δ*_H_ 7.32 (2H, d,* J* = 8.7 Hz, H-2′′/H-6′′) and 6.80 (2H, d,* J* = 8.7 Hz, H-3′′/H-5′′), as well as proton doublets showing* trans*-configuration at *δ*_H_ 7.37 (1H, d,* J* = 15.9 Hz, H-7′′) and 6.10 (1H, d,* J* = 15.9 Hz, H-8′′). Two aromatic carbon signals at *δ*_C_ 161.5 (C-4′′) and 127.1 (C-1′′) were correlated to two symmetric doublet proton signals and proton doublets with* trans*-configuration of* p*-coumaroyl moiety in the heteronuclear multiple bond connectivity (HMBC) spectrum. In addition, long-range HMBC correlations were, respectively, observed between anomeric proton (H-1′) and an oxygenated aromatic carbon at *δ*_C_ 143.1 (C-7), between a methoxy proton signal at *δ*_H_ 3.88 (3H, s) and C-6 carbon and a methoxy proton signal at *δ*_H_ 3.99 (3H, s) to C-8 aromatic carbon (Figure 1(b)). Moreover, downfield-shifted two oxygenated methylene signals at *δ*_H_ 4.39, 4.43 (H-6′) and *δ*_C_ 64.4 (C-6′) indicated that a carbonyl ester (C-9′′) group of* p*-coumaroyl moiety was attached with C-6′ of the glucose unit. On the basis of this data, the structure of compound** 1** was elucidated to be isofraxidin 7-*O*-(6′-*O*-*p*-coumaroyl)-*β*-glucopyranoside. The ^1^H and ^13^C NMR spectroscopic data (600 MHz, methanol-*d*_4_) for compound** 1** are shown in Table 1.

### 3.2. Effect of Compound** 1** on Melanin Production in B16F10 Melanocytes

**Figure 2:**
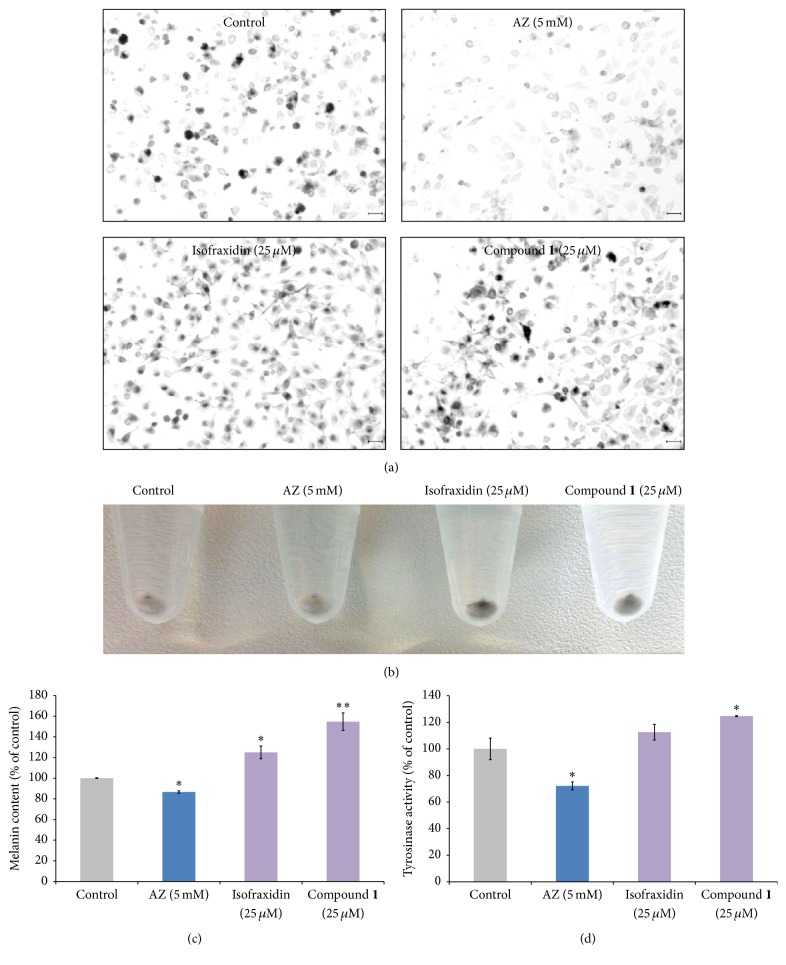
Effect of compound** 1** on melanin content in cultured B16F10 melanoma cells. (a) Cell images with or without the indicated treatment were taken using a digital camera (magnification 100x). Scale bar = 20 *μ*m. Untreated B16F10 show characteristic fibroblastic and spherical cell morphology. Isofraxidin and IFG treated B16F10 melanocytes show increased darkening of the cytoplasm compared to control cells after exposure to 25 *μ*M compound** 1** for 2 days. (b) Isofraxidin and compound** 1** treated B16F10 cell pellets are more pigmented compared to untreated cells. (c) B16F10 cells were treated with compound** 1** for 48 h at 37°C showing increased pigmentation in the melanocytes. (d) B16F10 melanocytes were treated with isofraxidin and compound** 1** at the indicated concentrations for 2 days. Compound** 1** also increased tyrosinase activity. ^*∗*^*p* < 0.05 compared to the untreated control. ^*∗∗*^*p* < 0.05 compared to isofraxidin treatment. AZ = azelaic acid.

To investigate whether compound** 1** regulates melanogenesis, melanin levels in B16F10 cells were measured after compound treatment. Along with compound** 1**, we also tested the effects of isofraxidin (7-hydroxy-6,8-dimethoxycoumarin), the parent compound of compound** 1**. The previously characterized melanogenic inhibitor, azelaic acid (AZ) [25], was used as a control. Addition of compound** 1** to melanocytes stimulated melanin production (Figures 2(a) and 2(b)). Compound** 1** significantly increased melanogenesis in a dose-dependent manner (Figure 2(c)). Moreover, melanocyte tyrosinase activity was also stimulated dose-dependently (Figure 2(d)). The parent compound, isofraxidin, also significantly increased melanin production, but not tyrosinase activity in B16F10 melanocytes (Figures 2(c) and 2(d)).

**Figure 3:**
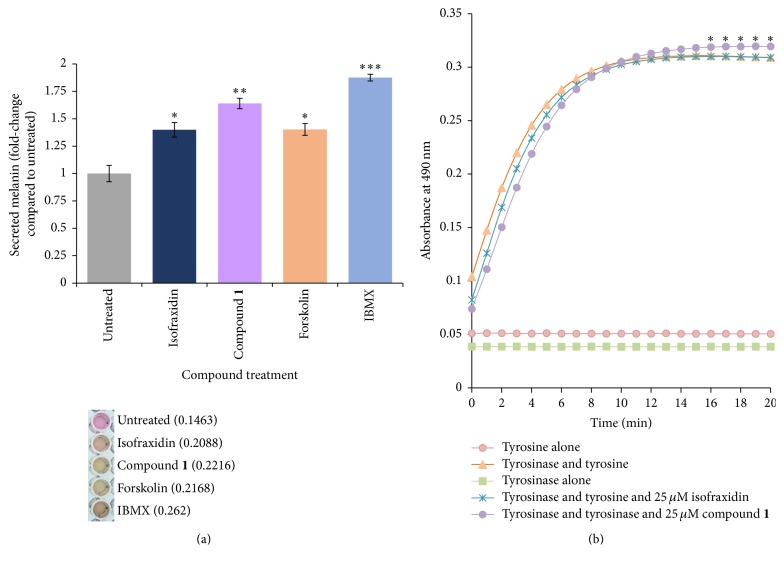
Compound** 1** increases melanin secretion and increases the activity of mushroom tyrosinase. (a) Effect of 25 *μ*M compound** 1** and 25 *μ*M isofraxidin on melanin secretion by B16F10 melanocytes. Culture media were collected after 60 h treatment. For comparison with known inducers of pigmentation, melanocytes were treated with 100 *μ*M IBMX or 5 *μ*M forskolin, as previously described [13, 14]. Error = SD; ^*∗*^*p* < 0.05 for increased melanin secretion compared to untreated cells; ^*∗∗*^*p* < 0.05 for increased melanin secretion compared to isofraxidin; ^*∗∗∗*^*p* < 0.05 for increased melanin secretion compared to compound** 1**. (b) Compound** 1**, but not isofraxidin, increases tyrosinase activity. Mushroom tyrosinase was used to measure tyrosinase activity in a cell-free assay. Error = SD; ^*∗*^*p* < 0.05 compared to tyrosinase plus tyrosine.

After treatment with melanogenic compounds, B16F10 cells are known to increase secretion of melanin into the culture media [15]. It was observed that compound** 1** treatment increased melanin secretion by the melanocytes (Figure 3(a)). This secretion was greater than that observed in isofraxidin treated melanocytes. Compared to known inducers of pigmentation, IBMX [14] produced a greater effect on pigmentation than compound** 1**, whereas forskolin [13] produced a lesser effect.

### 3.3. Compound** 1** Increases the Activity of Mushroom Tyrosinase

To further characterize the mechanisms by which compound** 1** increases melanocyte pigmentation, a cell-free mushroom tyrosinase assay was performed. It was observed that compound** 1** produced a significant increase in tyrosinase activity, which could be detected after 15 min of incubation (Figure 3(b)). In contrast, the parent compound, isofraxidin, did not affect tyrosinase activity.

### 3.4. Compound** 1** Upregulates the Expression of MITF and Tyrosinase in Melanocytes

**Figure 4:**
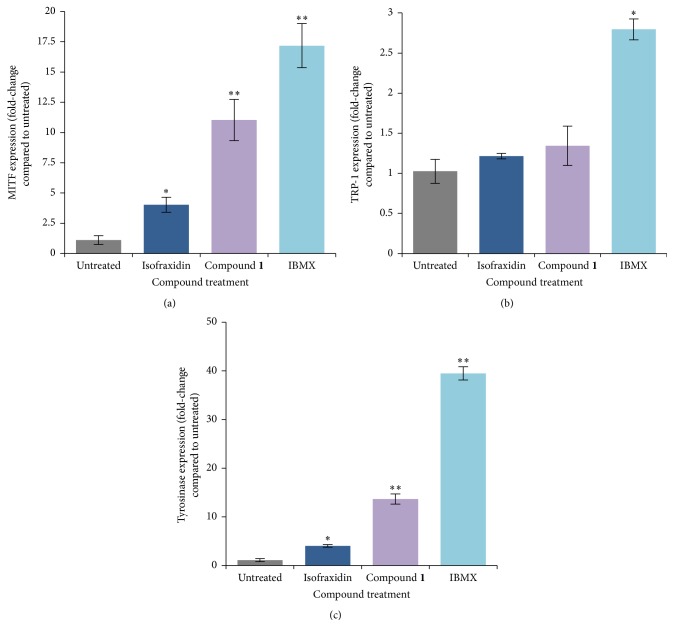
Real-time PCR analysis of MIFT, TRP-1, and tyrosinase gene expression in B16F10 melanocytes treated with 25 *μ*M isofraxidin, 25 *μ*M compound** 1** or IBMX for 48 h. (a) Expression of MIFT. ^*∗*^*p* < 0.05 compared to untreated cells; ^*∗∗*^*p* < 0.05 for increased expression compared to isofraxidin treated cells. (b) Expression of TRP-1. ^*∗*^*p* < 0.05 compared to untreated cells. (c) Expression of tyrosinase. ^*∗*^*p* < 0.05 compared to untreated cells; ^*∗∗*^*p* < 0.05 for increased expression compared to isofraxidin treated cells.

Three key regulatory genes for melanogenesis are microphthalmia-associated transcription factor (MITF), tyrosinase-related protein-1 (TRP-1), and tyrosinase [16, 26, 27]. Treatment of B16F10 melanocytes with compound** 1** for 48 h produced an increase in the expression of MITF and tyrosinase, but not TRP-1 (Figures 4(a)–4(c)). The increase in MITF and tyrosinase expression was greater than that observed after treatment with the parent compound, isofraxidin, at the same concentration.

### 3.5. Effect of Compound** 1** on Zebrafish Skin Pigmentation, Melanin Content, and Tyrosinase Activity

**Figure 5:**
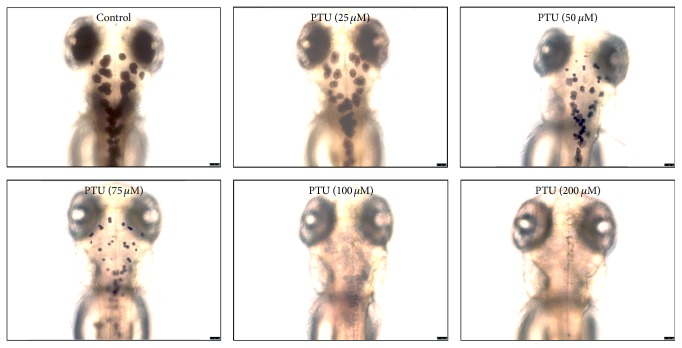
Dose-dependent effect of PTU on pigmentation in zebrafish embryos. Zebrafish were treated with PTU at concentrations of 25, 50, 75, 100, and 200 *μ*M. PTU treatment suppressed the production of black pigment spots (melanization) in the larva. Scale bar = 250 *μ*m.

To assess the effect of compound** 1** on pigmentation in vivo, we employed the zebrafish larvae model, which has proved useful for characterizing novel regulators of pigmentation [28]. Phenylthiourea (PTU), a known tyrosinase inhibitor [29], was tested as a positive control to assess in vivo alterations in pigmentation (Figure 5).

We found out that PTU dose-dependently abrogated pigmentation (Figure 5).

**Figure 6:**
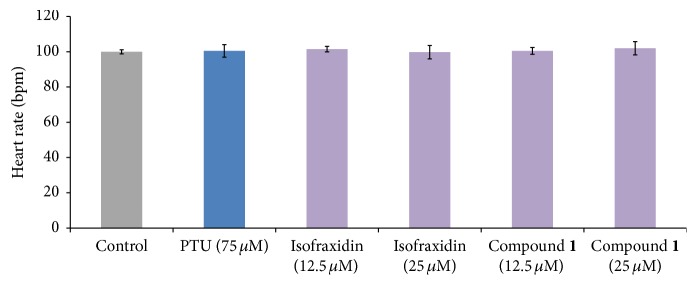
Effect of compound** 1** on average heart beat per minute (bpm) zebrafish embryos. The bpm of 48 hpf zebrafish embryos was assessed by visual examination.

**Figure 7:**
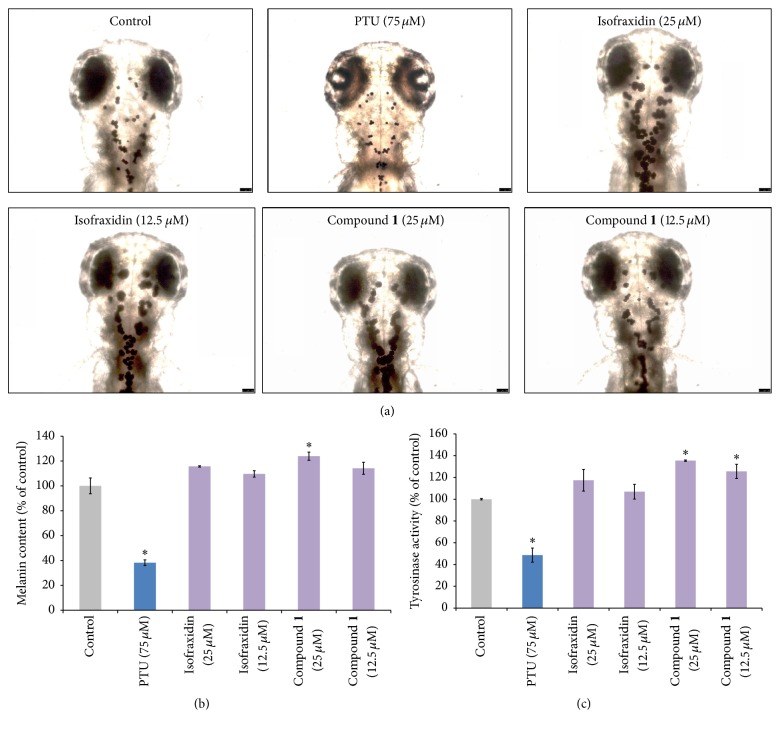
Effect of compound** 1** on pigmentation in zebrafish embryos. (a) 9 hpf embryos were treated with PTU and two concentrations of isofraxidin or compound** 1** (12.5 *μ*M and 25 *μ*M). Dorsal view of treated and untreated embryos at 72 hpf by stereomicroscope (magnification 100x). Isofraxidin and compound** 1** increased melanocyte pigmentation. Scale bar = 250 *μ*m. (b) Melanin content in zebrafish larvae treated with compound** 1** or isofraxidin. 25 *μ*M compound** 1** significantly increased melanin content in the zebrafish larvae. (c) Tyrosinase activity in zebrafish larvae treated with compound** 1** or isofraxidin. 12.5 *μ*M or 25 *μ*M compound** 1** significantly increased tyrosinase activity in the zebrafish larvae. ^*∗*^*p* < 0.05 compared to the untreated control larvae.

We evaluated the toxicity indicators, mortality rate, and heart rate [22, 30] in treated zebrafish embryos to identify the safe and effective concentration of compound** 1** (Figure 6). 9 hpf embryos were treated with compound. Greater than 90% of the treated embryos survived, which did not differ significantly from the control group. Based on this toxicity data, the effect compound** 1** on pigmentation in the zebrafish was tested at the 12.5 *μ*M and 25 *μ*M dose (Figure 7(a)). Compound** 1** and isofraxidin were observed to increase melanocyte size in the zebrafish larvae. To quantify melanin content, 40 zebrafish larvae were treated with compound** 1** or isofraxidin. It was observed that compound** 1** increased melanin content at the 25 *μ*M dose (Figure 7(b)). The tyrosinase activity was carried out in treated zebrafish larvae (Figure 7(c)). It was observed that the 12.5 and 25 *μ*M doses of compound** 1** increased tyrosinase activity (Figure 7(c)). Overall, these findings indicate that the hyperpigmentation effect of compound** 1** is mediated through the stimulation of tyrosinase activity.

### 3.6. Effect of Compound** 1** on Zebrafish Melanocyte Pigmentation

**Figure 8:**
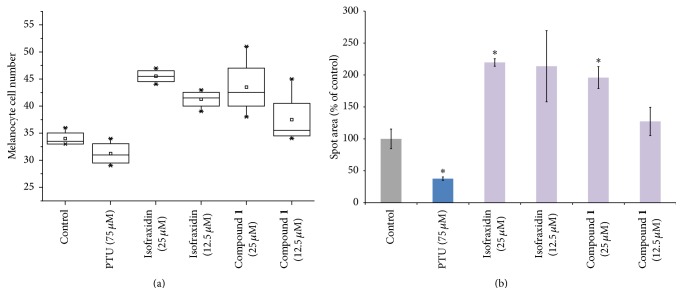
Effect of compound** 1** on melanocyte numbers and surface area in zebrafish embryos. (a) Box and whisker plot of melanocyte numbers in the head region in embryos treated with PTU, isofraxidin, or compound** 1** at 48 hpf. (b) Surface area of melanocytes in the treated and untreated embryos at 48 hpf. ^*∗*^*p* < 0.05 compared to the untreated larva.

To further characterize the effect of compound** 1** on melanocyte pigmentation, we measured melanocyte development in compound treated and untreated zebrafish skin. Zebrafish melanocytes develop from melanoblasts at about 25 hpf, and by 60 hpf the larval pigment pattern is established [31]. We observed that zebrafish embryos treated with isofraxidin or compound** 1** showed increased numbers of visible melanocytes compared to the control embryo (Figure 8(a)). Moreover, isofraxidin or compound** 1** also produced an increase in the melanocyte area in the treated embryos (Figure 8(b)).

## 4. Discussion

Our study describes the discovery of compound** 1** as a hyperpigmentation agent isolated from* A. capillaris* that is effective in an in vivo system. Compound** 1 **increased pigmentation without producing toxicity.

Compound** 1** produced greater effects on melanin production and secretion in melanocytes compared to the parent compound, isofraxidin (Figures 2(c) and 3(a)). To our knowledge, there is no previous report that isofraxidin can increase pigmentation. Our results show that isofraxidin can increase melanin content in melanocytes (Figure 2(c)). However, tyrosinase activity was not affected (Figure 2(d)). In contrast, compound** 1** significantly increased melanin content in melanocytes compared to isofraxidin (Figures 2(c) and 2(d)). A potential mechanism to explain the stronger pigmentation effect of compound** 1** is the higher induction of pigmentation gene expression (MITF and tyrosinase) relative to isofraxidin (Figures 4(a) and 4(c)). In addition, compound** 1** increased the activity of tyrosinase in the cell-free assay, which was not observed after isofraxidin treatment (Figure 3(b)). In the zebrafish pigmentation system, both isofraxidin and compound** 1** increased melanocyte number and area (Figures 8(a) and 8(b)). However, only compound** 1** produced significant increases in melanocyte accumulation and tyrosinase activity in this animal model (Figures 7(b) and 7(c)) indicating that compound** 1** is a more potent inducer of pigmentation compared to isofraxidin.

In summary, we have discovered that compound** 1** is a novel, nontoxic pigmentation enhancer that is effective in vivo. Compound** 1** targets the expression of MITF and tyrosinase expression/activity. These results indicate that this natural product can be further developed as a therapeutic for treating hypopigmentation disorders.

## Figures and Tables

**Table 1:** ^1^H and ^13^C NMR spectroscopic data (600 MHz, methanol-*d*_4_) for isofraxidin 7-*O*-(6′-*O*-*p*-coumaroyl)-*β*-glucopyranoside.

Position	*δ*_H_ (*J* in Hz)	*δ*_C_	HMBC^a^
2	—	162.7	
3	6.16 1H, d (9.6)	115.7	C-2, 4a
4	7.64 1H, d (9.6)	145.7	C-2, 5
4a	—	116.9	
5	6.90 1H, s	105.9	C-4, 6, 7, 8a
6	—	151.8	
7	—	143.1	
8	—	142.7	
8a	—	144.0	
1′	5.20 1H, d (7.5)	103.9	C-7
2′	3.55 1H, dd (7.5, 9.0)	75.7	C-1′, 3′,
3′	3.47 1H, t (9.0)	77.9	C-2′, 4′
4′	3.40 (1H, t, 9.0)	72.2	C-3′, 5′
5′	3.50 1H, m	75.9	C-4′
6′	4.39 1H, dd (11.4, 7.2)	64.4	C-4′, 5′
	4.34 1H, dd (11.4, 2.0)		
1′′	—	127.1	
2′′/6′′	7.32 2H, d (8.7)	131.3	C-4′′
3′′/5′′	6.80 2H, d (8.7)	117.1	C-1′′, 4′′
4′′	—	161.5	
7′′	7.37 1H, d (15.9)	146.5	C-1′′, 2′′/6′′, 9′′
8′′	6.10 1H, d (15.9)	114.9	C-7′′, 9′′
9′′	—	168.7	
6-OMe	3.88 3H, s	57.2	C-6
8-OMe	3.99 3H, s	62.5	C-8

^a^HMBC correlations for compound **1**.

## Abbreviations

^1^H NM: Proton nuclear magnetic resonance
^13^C NMR: Carbon nuclear magnetic resonance
*A. capillaris*: * Artemisia capillaris* Thunberg
ACN: Acetonitrile
AZ: Azelaic acid
DMSO: Dimethylsulfoxide
Hpf: Hours postfertilization
HPLC: High performance liquid chromatography
IBMX: 3-Isobutyl-1-methylxanthine
L-DOPA: * L*-*3*,*4*-Dihydroxyphenylalanine
MeOH: Methyl alcohol
MITF: Microphthalmia-associated transcription factor
NaOH: Sodium hydroxide
PTU: Phenylthiourea
TRP-1: Tyrosinase-related protein-1.
